# Risk factors for Covid-19 severity and fatality: a structured literature review

**DOI:** 10.1007/s15010-020-01509-1

**Published:** 2020-08-28

**Authors:** Dominik Wolff, Sarah Nee, Natalie Sandy Hickey, Michael Marschollek

**Affiliations:** Peter L. Reichertz Institute for Medical Informatics of TU Braunschweig and Hannover Medical School, Hannover, Germany

**Keywords:** Covid-19, SARS-CoV-2, Review, Risk factors, Population at risk

## Abstract

**Purpose:**

Covid-19 is a global threat that pushes health care to its limits. Since there is neither a vaccine nor a drug for Covid-19, people with an increased risk for severe and fatal courses of disease particularly need protection. Furthermore, factors increasing these risks are of interest in the search of potential treatments. A systematic literature review on the risk factors of severe and fatal Covid-19 courses is presented.

**Methods:**

The review is carried out on PubMed and a publicly available preprint dataset. For analysis, risk factors are categorized and information regarding the study such as study size and location are extracted. The results are compared to risk factors listed by four public authorities from different countries.

**Results:**

The 28 records included, eleven of which are preprints, indicate that conditions and comorbidities connected to a poor state of health such as high age, obesity, diabetes and hypertension are risk factors for severe and fatal disease courses. Furthermore, severe and fatal courses are associated with organ damages mainly affecting the heart, liver and kidneys. Coagulation dysfunctions could play a critical role in the organ damaging. Time to hospital admission, tuberculosis, inflammation disorders and coagulation dysfunctions are identified as risk factors found in the review but not mentioned by the public authorities.

**Conclusion:**

Factors associated with increased risk of severe or fatal disease courses were identified, which include conditions connected with a poor state of health as well as organ damages and coagulation dysfunctions. The results may facilitate upcoming Covid-19 research.

## Introduction

In the end of 2019, a novel respiratory disease, the coronavirus disease 2019 (Covid-19), occurred. The pathogen causing the disease was identified by next-generation sequencing as a novel coronavirus closely related to the SARS-coronavirus discovered in 2003 [1]. According to the WHO guidelines [2], this novel coronavirus was named severe acute respiratory syndrome coronavirus 2 (SARS-CoV-2). First cases of Covid-19 were reported from the Chinese city Wuhan located in the province Hubei in December 2019 [3]. The disease is spreading worldwide and was classified as a pandemic by the WHO in March 2020 [4]. The virus is transmissible from human to human [5] and the number of infected people increases at an exponential rate, exceeding 1 mio. cases on 02.04.2020 and 1.5 mio. cases in 184 countries only a week later [6, 7]. At various disease hotspots such as New York, the health care system reaches its limits.

For diagnosis, the virus is mainly detected by real-time quantitative polymerase chain reaction (rt-PCR) in throat swabs [8, 9]. Due to limited test capacities, which require a special equipped laboratory, patients showing symptoms are tested only. On the onset of Covid-19 typical symptoms are fever, cough, myalgia and fatigue, while headache, sputum production, hemoptysis and diarrhea are less common. In the course of disease a subset of patients show pneumonia with abnormal findings on chest CT [10]. Severe cases are transferred to an intensive care unit (ICU) and frequently require artificial ventilation. The disease’s case fatality rate is estimated between 3.4% and 11% [11]. Although, it depends to a large extent on the number of tests carried out as well as the quality and occupancy rate of local health care.

Until a vaccine is available, an increase in the number of infections must be expected and if not being controlled Covid-19 will exceed the limits of health care systems. Since some groups appear to be at higher risk of serious disease progression and increased mortality, they should be given special protection against an infection. This is particularly important in the context of the much-discussed relaxation of restrictions, such as the prohibition of contact. To identify these vulnerable groups, the risk factors for severe and fatal disease progression must be found. Additionally, the identification of risk factors can contribute to research into the pathophysiological processes of Covid-19 from which possible treatment strategies can be developed. However, information on this is scattered and based on rather small studies. For connecting these, this publication describes a structured literature review on the risk factors of Covid-19 for severe and fatal disease courses. Additionally, the review’s results are compared to the risk factors mentioned by four public authorities.

## Methods

Publications of interest describe clinical studies on Covid-19 identifying factors for increased risks of severe or fatal disease courses. The review focusses on studies whose patients were diagnosed positive by rt-PCR. The diagnosis by rt-PCR shows a low false-positive rate, but is criticized for a quite high false-negative rate [12, 13]. The inclusion of rt-PCR diagnoses only reduces the number of false diagnoses to a minimum. Since the disease is new and has only been present since December 2019, the search is carried out on PubMed as well as on the Covid-19 Open Research Dataset (CORD-19) [14] containing mostly yet unpublished publications, so called preprints.

To identify publications of interest, MESH Terms and synonyms for Covid-19 and risk factors are combined leading to the following search term:

- ("risk factor" OR "determinant" OR "disposition" OR "increased risk" OR "population at risk" OR "health risk behavior") AND ("covid-19" OR "sars-cov-2" OR "covid19" OR "2019-nCov" OR "severe acute respiratory syndrome coronavirus 2 " OR "covid 19").
- Furthermore, the search is limited to the English language. It was performed on PubMed on 25.03.2020 and was updated on 17.04.2020. Search results were documented as file export including search term and date.

### CORD-19

The Covid-19 Open Research Dataset (CORD-19) was created by the Allen Institute for AI in partnership with the Chan Zuckerberg Initiative, Georgetown University’s Center for Security and Emerging Technology, Microsoft Research, and the National Library of Medicine-National Institutes of Health, in coordination with the White House Office of Science and Technology Policy. It is freely available and updated weekly. The data provided is intended to facilitate the application of natural language processing to generate new insights in support of the fight against Covid-19. The dataset contains more than 51,000 scholarly articles on SARS-CoV-2 and related coronaviruses such as SARS-CoV and the Middle East Respiratory Syndrome (MERS) Coronavirus including over 40,000 full texts [14]. Beside these documents, a file containing the publications’ metadata, is provided. It contains information such as title, DOI, PubMed ID and the abstract, but is not limited to these. In a first step the metadata is preprocessed and a keyword search is performed to identify publications of interest. Afterwards, the typical literature review procedure is carried out, including screening of title and abstract for eligibility and accessing the full texts.

For preprocessing of the data, a simple algorithmic pipeline was applied to the metadata file. First, information of interest (PubMed ID, title, abstract, availability of the full text) are extracted. In the next steps, all articles without a full text, with abstracts shorter than 20 words and with abstracts in a different language than English are excluded. Afterwards, the abstracts and keywords are transformed to lower case characters to perform an algorithmic keyword search analog to the above-mentioned search term. The search was performed on 25.03.2020 and updated on 21.04.2020.

### Analytical methods

In a first step the identified publications’ titles and abstracts are screened for eligibility. For publications describing literature reviews or meta-studies, the references are checked for eligibility. Full texts of suitable publications are then analyzed regarding the inclusion criteria. Both steps were executed by multiple researchers.

The analysis centers on the identification of risk factors for severe and fatal disease progression. Risk factors found are categorized into lifestyle factors, demographic factors, pre-existing comorbidities, due to Covid-19 developed comorbidities, symptoms and clinical factors. Additionally, information on the studies are extracted including study size, location, duration, mono- or multicentricity and whether the data collected is available. For characterization of the articles, the publication status is recorded. To ensure the quality of preprints, the comprehensibility and correctness of the study design and statistical analysis is evaluated. In the event of uncertainty, the decision is to exclude the record. P-values smaller than 0.05 are regarded as significant.

## Results

**Fig. 1:**
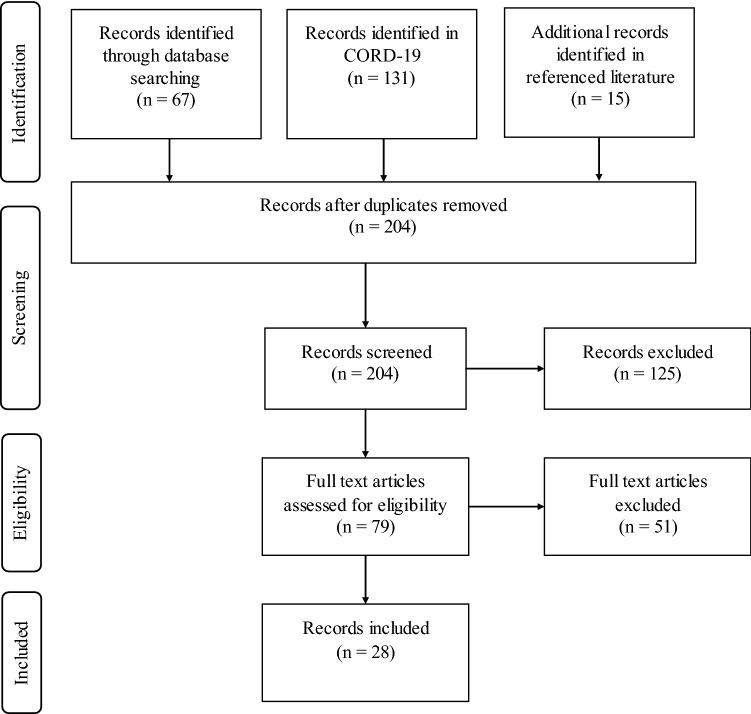
Overview of the publication selection process

In the search 213 papers were identified (67 PubMed, 131 CORD, 15 referenced literature). After the removal of duplicates 204 records were screened based on title and abstract. In this step, 125 records were excluded. The remaining records’ full texts were assessed and 51 records were excluded for not describing risk factors backed by a clinical study or not diagnosing patients by rt-PCR. Thus 28 records were included (see Fig. 1).

**Fig. 2:**
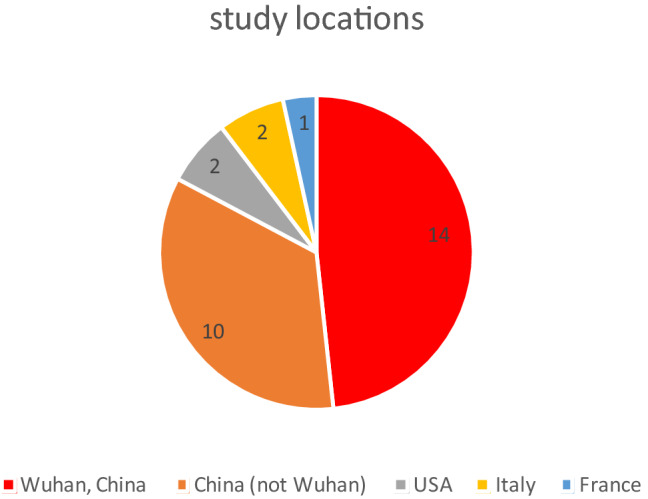
Number of studies found by location

Table 1 shows a description of the studies found in the records included. From the 28 included records 17 are published and 11 are preprints. The studies described took place at the end of 2019 and in the first months of 2020. The last inclusion of a patient was on 05.04.2020 in [40]. Most studies found were conducted in China (*n* = 24), while the remaining five studies were conducted in Italy, France and the USA (see Fig. 2). Eighteen studies were carried out at a single place, while twelve studies were multicentric, involving between 2 and 575 hospitals. The patient numbers of the individual studies range between 25 and 62,843, with small studies with up to 200 patients being the norm.

**Table 1:** Overview of the records included

Reference	Review state	Study size	Study location	Centricity (# of centers)	Study duration	Data published
[15]	Published	25	Wuhan, China	Monocentric	14.01.2020–13.02.2020	Data published
[16]	Published	138	Wuhan, China	Monocentric	01.01.2020–28.01.2020	Synoptic table
[17]	Published	140	Wuhan, China	Monocentric	16.01.2020–03.02.2020	Synoptic table
[18]	Preprint, not peer reviewed	128	Xiangyang, China	Monocentric	01.01.2020–16.02.2020	Synoptic table
[19]	Preprint, not peer reviewed	198	Shanghai, China	Monocentric	20.01.2020–15.02.2020	Full dataset on request
[20]	Preprint, not peer reviewed	141	Changsha, China	Multicentric (2)	17.01.2020–01.02.2020	Synoptic table
[21]	Published	43; 1056	Wuhan, China	Mono-, multicentric (6)	29.01.2020–15.02.2020	Full dataset on request
[22]	Preprint, not peer reviewed	710	Wuhan, China	Multicentric (3)	28.01.2020–11.02.2020	Synoptic table
[23]	Published	383	Wuhan, China	Monocentric	02.01.2020–01.03.2020	Synoptic table
[24]	Published	245	Wuhan, China	Monocentric	01.01.2020–29.02.2020	Synoptic table
[25]	Preprint, not peer reviewed	1902	Wuhan, China	Multicentric (3)	28.01.2020–08.03.2020	Synoptic table
[26]	Preprint, not peer reviewed	355	Wuhan, China Fuyang, China	Multicentric (2)	?	Synoptic table
[27]	Published	54	Stanford, USA	Monocentric	Until 16.03.2020	Full dataset on request
[28]	Preprint, not peer reviewed	258	Wuhan, China	Monocentric	29.01.2020–12.02.2020	Full dataset on request
[29]	Preprint, not peer reviewed	84	Yongchuan, China	Monocentric	21.01.2020–02.03.2020	Full dataset on request
[30]	Preprint, not peer reviewed	62,843	complete Italy	Multicentric (?)	Until 24.03.2020	Synoptic table
[31]	Published	4,103	New York City, USA	Multicentric (4)	01.03.2020–01.04.2020	Synoptic table
[32]	Published	701	Wuhan, China	Monocentric	28.01.2020–11.02.2020	Full dataset on request
[33]	Published	323	Wuhan, China	Monocentric	08.01.2020–20.02.2020	Synoptic table
[34]	Published	1590	complete China	Multicentric (575)	Until 31.01.2020	Synoptic table
[35]	Preprint, not peer reviewed	564	Hunan, China	Multicentric (9)	17.01.2020–28.02.2020	Full dataset on request
[36]	Preprint, not peer reviewed	36	Shenyang, China	Multicentric (3)	26.01.2020–15.02.2020	Synoptic table
[37]	Published	52	Wuhan, China	Monocentric	12.2019–26.01.2020	Full dataset on request
[38]	Published	54	Hubei, China	Monocentric	?	Synoptic table
[39]	Published	1591	Lombardy, Italy	Multicentric (72)	20.02.2020–18.03.2020	Synoptic table
[40]	Published	124	Lille, France	Monocentric	27.02.2020–05.04.2020	Synoptic table
[41]	Published	30	Huizhou, China	Monocentric	01.2020–02.2020	Synoptic table
[42]	Published	174	Wuhan, China	Monocentric	10.02.2020–29.02.2020	Synoptic table

### Risk factors for severity

Risk factors for disease severity were identified in 20 records, which are described in Table 2. Smoking [33], a higher body mass index (obesity) [40] and a longer waiting time to hospital admission [19, 20] are lifestyle factors related to a higher risk for disease severity. The most frequently mentioned demographic factor increasing the risk for a severe course of disease is higher age [16, 17, 19, 21, 27, 30, 31, 33, 35, 41, 42], followed by male gender [19, 21, 25], post menopausality [25] and higher age in females [25]. Some publications specify the age for increased risk as > 64 [31] or > 65 [33] years. The most common pre-existing comorbidities are hypertension [16, 19, 27, 35, 40, 42] and diabetes [16, 28, 33, 35, 40, 42] with six records each, followed by cardiovascular disease with three records [16, 19, 35]. Occasionally, correlations of the severity and cerebrovascular disease [16], chronic obstructive pulmonary disease [35], chronic renal disease [35] or tuberculosis [36] were found. For eight comorbidities developed during the Covid-19 infection a significant impact on disease severity was found. These are organ failure [19], immunological dysfunction [19], acute liver injury [26], hypoproteinemia [26], Acute Respiratory Distress Syndrome (ARDS) [36], severe pneumonia [42], uncontrolled inflammation response [42] and hypercoagulable state [42]. With nine mentions, the most common abnormal clinical factor is decreased lymphocytes, followed by an increased d-dimer level (six records), increased leucocytes (four records), increased neutrophil count (four records), increased aspartate aminotransferase (AST) (four records), increased c-reactive protein (CRP) (four records), increased alanine aminotransferase (ALT) (three records) and low oxygen saturation (three records). Increased blood urea nitrogen (BUN), decreased thrombocytes, increased CT severity score and increased interleukin 6 (IL-6) level are each identified as risk factors for severity in two records. There are 23 other clinical features such as decreased blood sodium or decreased erythrocytes count each mentioned in one record only (see Table 2). In addition to the factors already mentioned, the symptoms fever (> 38.5 °C) [18, 35] and dyspnea [18, 35] are associated with severe disease progression.

**Table 2:** Listing of the factors found that influence the severity of the disease

Reference	Lifestyle factors	Demographic factors	Preexisting comorbidities	Developed comorbidities	Clinical factors	Symptoms
[16]		Higher age	Hypertension Diabetes Cardiovascular disease Cerebrovascular disease		Increased white blood cell count Increased neutrophil counts Increased d-dimer level Increased creatine kinase level Increased creatine level Increased blood Urea nitrogen Increased aspartate aminotransferase Increased alanine aminotransferase Decreased lymphocyte	
[17]		Higher age			Increased leukocytes Decreased lymphocyte percentage Increased d-Dimer Increased C-reactive Protein Increased Procalcitonin (PCT)	
[18]					increased white blood cell count Increased CT glass opacity decreased lymphocytes Decreased platelets Increased alanine transaminase Increased aspartate transaminase Increased C-reactive protein	
[19]	Longer waiting time to admission	Higher age male gender	Cardiovascular disease	Organ failure Immunological dysfunction	Decreased lymphocytes Increased neutrophils increased prothrombin time Increased activated partial thromboplastin time Increased fibrinogen increased d-dimer Decreased blood sodium Decreased calcium	Fever > 38.5 °C Dyspnea
[20]	Longer waiting time to admission	Higher age	Hypertension		Decreased lymphocyte count Increased neutrophil-to-lymphocyte ratio (NLR) Increased C-reactive protein Increased CT severity score	
[21]		Higher age Male gender				
[25]		Post-menopausality Higher age of females Male gender			Increased Interleukin 6 Increased Interleukin 8 E2 and AMH are negatively correlated	
[26]				Acute liver injury Hypoproteinemia	Elevated total bilirubin, elevated direct bilirubin Elevated indirect bilirubin, Elevated ALT Elevated AST Decreased total protein Decreased albumin Decreased albumin per globulin ratio	
[27]		Higher age	Hypertension		Low presenting oxygen saturation	
[28]			Diabetes			
[29]		Higher age				
[30]		Higher age				
[31]		Age > 64			Admission oxygen saturation < 88% first d-dimer > 250 First C-reactive protein > 200 SpO2 < 88 Procalcitonin > 0.5 Troponin < 0.1 C-reactive protein > 200	
[33]	Smoking	Age > 65	Diabetes		Abnormally higher hypersensitive troponin I (> 0.04 pg/mL) Leucocyte count > 10 × 10^9^/L neutrophil count > 75 × 10^9^/L	
[35]		Higher age	Hypertension Diabetes Cardiovascular disease Chronic obstructive pulmonary disease Chronic renal disease		Increased aspartate aminotransferase Increased blood urea nitrogen d-dimer ≥ 0.05 mg/L Increased lactose dehydrogenase Worse lung CT score Decreased lymphocyte count	Presented with fever Presented with shortness of breath
[36]			Tuberculosis	Acute respiratory distress syndrome		
[37]					Lymphocytopenia	
[40]	Higher BMI		Diabetes and hypertension (dependent with obesity)		Decreased blood oxygen saturation Need for oxygen support therapy for at least 6 L/min	
[41]		Higher age			Increased platelet‐to‐lymphocyte ratio at platelet peak Decreased lymphocyte count Higher platelet peak	
[42]		Higher age	Diabetes Hypertension	Severe pneumonia Uncontrolled inflammation responses Hypercoagulable state	Elevated Interleukin 6 Elevated C-reactive protein Elevated serum ferritin Elevated coagulation index Elevated d-dimer Decreased count of lymphocytes Higher absolute count of neutrophils Decreased erythrocytes counts Decreased hemoglobin	

### Risk factors of fatal disease courses

Thirteen records describe risk factors for fatal Covid-19 disease courses. They are listed in Table 3. The most common identified risk factor is high age with eight denominations. The other demographic factor influencing Covid-19 mortality is male gender, which was found significant in three records. Furthermore, pre-existing comorbidities frequently show an influence in the publications included. Most common with three mentions each are hypertension, diabetes and coronary heart disease. Cardiovascular diseases are found significant in two records. Seven other pre-existing diseases were each significant in one record, including acute liver injury, kidney disease, chronic illnesses and cerebrovascular disease. For comorbidities developed during the infection, kidney injuries (four records), heart injuries (three records) and liver injuries (two records) are mentioned most often. Other developed complications are cardiac death, acute respiratory distress syndrome, hospital acquired infections, thrombocytopenia and hypoxemia. Only one record identified a symptom, dyspnea, as a risk factor. The most common clinical factors associated with mortality are increased creatinine (four records), increased c-reactive protein (CRP), increased procalcitonin (PCT), decreased lymphocytes and increased blood urea nitrogen (BUN) (three records each). Other clinical factors associated with fatal disease courses include increased neutrophils, increased leucocytes or increased d-dimer but are not limited to these. For the full list of clinical factors found in the records please refer to Table 3.

**Table 3:** Listing of the factors found with an influence on fatal disease courses

Reference	Lifestyle factors	Demographic factors	Preexisting comorbidities	Developed comorbidities	Clinical factors	Symptoms
[15]				Heart damage Kidney damage Liver damage	Decrease albumin Increased PCT Increased neutrophils Increased C-reactive protein Increased cTnI Increased d-Dimer Increased LHD Decreased lymphocyte level	
[21]		Higher age Male gender				
[22]		Age > 65 years		Kidney impairment	Leucocyte count > 4 × 10^9^/L Lymphocyte < 1.5 × 10^9^/L Increased serum creatinine baseline Increased serum creatinine peak Increased blood urea nitrogen (BUN) Increased proteinuria Increased hematuria	
[23]				Thrombocytopenia	Decreased platelet count (40% decrease in mortality risk for every 50 × 10^9^/L increase) Dynamic change of platelets	
[24]	Higher BMI	Higher age	Hypertension Diabetes Coronary heart disease		Neutrophil to lymphocyte ratio (NLR) 8% higher risk per unit increase Respiratory rate > 30 bpm Increased neutrophil Increased ALT Increased creatinine Increased prothrombin Increased C-reactive protein Increased procalcitonin	
[26]			Hypoproteinemia Cholestasis Acute liver injury		CT abnormalities Patchy shadows Ground glass opacities Consolidation Interlobular septal thickening Higher CT value	
[28]			Diabetes			
[30]		Higher age Male gender				
[32]			Kidney disease	Acute kidney injury	Elevated baseline serum creatinine Elevated baseline blood urea nitrogen (BUN) Proteinuria Hematuria	
[34]		Age > 65	Coronary heart f Disease Cardiovascular disease		PCT > 0.5 ng/ml AST > 40U/l	Dyspnea
[37]		Higher age	Chronic illness Cerebrovascular disease	Acute Respiratory Distress Syndrome Hospital acquired infection organ function damage (kidney, cardiac, liver) Hypoxemia	Low ratio partial pressure oxygen (PaO_2_) to F_i_O_2_	
[38]		Higher age	Hypertension Coronary heart disease	Heart injury Cardiac death	Increased NT-proBNP Increased myohemoglobin Increased CK-MB Increased hs-TnI Increased blood urea Increased creatinine Increased white blood cell count Increased CRP Increased procalcitonin Decreased lymphocyte Higher diastolic blood pressure	
[39]		Higher age Male gender	Hypertension Cardiovascular disease Hypercholesterolemia Diabetes			

Typically, a severe course of the disease occurs before the death of a Covid-19 patient. Of course, this is not true for all fatal courses, but it should be true for most of them and therefore be visible in the statistical significance. The risk factors for fatal courses should be approximately a subset of the factors for severe courses. Therefore, risk factors for fatal disease progression, which are not mentioned for severe disease progression, are of particular interest. For pre-existing comorbidities these are coronary heart disease, hypoproteinemia, cholestasis, acute liver injury and hypercholesterolemia, while hypoproteinemia and acute liver injury are also mentioned as developed comorbidities in severe courses. Developed comorbidities found with an influence on fatal courses but not on severe courses are heart damage, kidney damage, thrombocytopenia, hospital acquired infections, hypoxemia and cardiac death.

### Disease specific laboratory values

Some laboratory values found are predictive for specific diseases. Most common are markers for liver, renal and heart function. Increased ALT, AST, lactic acid, procalcitonin, total, direct and indirect bilirubin as well as decreased albumin indicate liver injuries [43]. The same applies for increased blood urea nitrogen and creatinine as well as proteinuria and hematuria for renal injuries [44]. Heart specific markers found in the publications are increased creatine kinase, troponin C and myohemoglobin levels as well as a decreased platelet count [45]. It is also noticeable that an increased number of coagulation factors such as decreased platelets, increased d-dimer level and increased fibrinogen [46] as well as inflammatory parameters such as c-reactive protein [47] and increased leucocyte level are associated with severity and fatality.

## Discussion

This review shows a high exclusion rate (176/204), which is mainly caused by including studies identifying Covid-19 infections explicitly by rt-PCR only. However, a high significance of the results can be guaranteed, as other diseases, such as bacterial pneumonia, are clearly excluded by the rt-PCR identification. Identification by rt-PCR has also become the standard diagnostic procedure. Nevertheless, it must be assumed that a selection bias exists in the results obtained, since most studies do not provide a representative sample. Among other things, differences in the recruitment rate and different test procedures have an influence on this. Relying on rt-PCR based studies only enhances this effect. A portion of the included papers are preprints, which were not yet peer-reviewed. This allows early scientific results to be incorporated into the analysis performed in this record. Even though a quality review by the authors has been carried out, which includes the comprehensibility and correctness of the study design as well as the statistical analysis, the results of these preprinted studies should be used with caution in further decisions concerning Covid-19. The publication status of the preprints should be reviewed at a later date.

Most records included describe studies carried out in China. This is presumably since the disease first broke out in China and spread around the world only within the next weeks and months. Data from Chinese Covid-19 patients is available earlier and can therefore be analyzed and published earlier. When comparing the data on the level of the number of patients or facilities, a different picture arises. The majority of patients included in this review are from Italy (64,434), followed by China (7656), USA (4157) and France (1715). Therefore, statements for specific ethnicity cannot be made and the results should be generally interpreted. It should be noted that a doubling of patients between studies cannot be excluded. This is especially relevant for some of the Chinese publications, which show an overlapping of the author list and the recruitment time, potentially reducing the real number of patients. Based on the number of publications and the number of patients, it seems that Italy is trying to centralize research on Covid-19, while China tends to produce smaller individual studies. Both approaches have advantages and disadvantages. Individual studies can deliver results more quickly and be transferred to the community, while centralization allows linking the data so that statements of higher quality can be made. The rather small proportion of studies from the USA and Europe could be linked to the date the search was carried out and the course of the disease’s global spread. We expect to see more studies from these countries as well as other Asian countries in the future. Concerning the number of patients, publication [30] is particularly noteworthy as it summarizes all cases in Italy until the beginning of March. Unfortunately, the data of this study are not published as a complete data set. However, the publication rate of the collected data is quite high among the studies included as data of eight studies is publicly available or available upon request.

Since no special drug for treating Covid-19 exists, a longer waiting time to hospital admission is an eye-catching risk factor for severity. This indicates that the treatment of symptoms in an early disease stage can be effective and positively influence the disease’s course. Regarding other demographic and lifestyle factors found interdependencies are very likely. First, younger women will not be menopausal and therefore post menopausality is equivalent to higher age, which is the most named risk factor in this analysis. Second, with higher age comorbidities are getting more likely to be present while the immune systems is getting weaker [48]. This means higher age (approximately > 60 years) is very likely correlated with comorbidities such as hypertension, cardiovascular diseases and diabetes, which are the most common comorbidities in this review. Third, hypertension is a risk factor for cardiovascular diseases [49] and, since cardiovascular disease appear to be a risk factor for Covid-19, hypertension is a risk factor for Covid-19 as well. Although multivariate regression analyses are performed in 16 records, those dependencies could not be confirmed. More research and testing on interdependence of risk factors should to be carried out.

Typically, for a disease that primarily affects the lungs, it would be expected that lung-damaging behaviors, such as smoking, or pre-existing lung diseases increase the risk for severe courses. It is very striking that smoking shows a significant influence in only one publication as well as lung diseases not being commonly listed as risk factors for either severe or fatal disease progression. This may be related to the fact that the definition of a severe disease course is based on severe pneumonia and is therefore not listed. However, other pre-existing lung diseases such as chronic obstructive pulmonary disease (COPD) are only named in a few records. For nicotine on the other hand the ability to downregulate the ACE-2 level, which is a functional receptor for SARS-CoV-2 [1], was shown [50]. Furthermore, a mouse study [51] suggests that nicotine protects against acute inflammation in lung tissue by activating nicotinic acetylcholine receptors on immune cells which inhibits the release of pro-inflammatory cytokines. However, nicotine’s influence on the course of Covid-19 needs further research.

Regarding disease predictive clinical factors liver, renal and heart damage are most common, which are also present as comorbidities associated with increased risk. It can be assumed that Covid-19 damages these organs and pre-existing damages further promote the impact. Eleven records found coagulation factors positively associated with severity or fatality but only two ([23] and [42]) mention them directly in the publication. Therefore, the influence of coagulation disorders and their treatments on the course of the disease should be further examined. It is also possible that the above-mentioned organ damage is promoted or triggered by Covid-19 induced coagulation disorders. A newer pathological study with twelve deceased Covid-19 patients found high incidence of thromboembolic events suggesting an important role of Covid-19 - induced coagulopathy. Even more, 5 of the 12 patients showed high viral RNA titers in the liver, kidney, or heart [52]. In addition, laboratory values indicating heart, liver and renal damages are significant in the included records for fatal disease courses but not for severe ones. This suggests that organ damages, specifically heart, kidney and liver damages, are symptoms occurring in the late phase of Covid-19 infections.

For some risk factors found, it cannot be entirely excluded that they are manifestations of the disease itself and not real risk factors. This is especially the case for risk factors that are very close to the clinical picture of Covid-19, such as low oxygen saturation or ARDS. For cardinal symptoms of a severe disease, statistical significance is very likely to be found. Even if a significant influence on the severity of the disease has been found in several studies, it must be understood that causality does not necessarily follow from statistical significance.

Still, there are limitations to this review. Due to the exclusive focus on PCR diagnostics it is possible that some important factors are dismissed, which were found in studies relying on clinical diagnostics. However, the focus on PCR diagnosis increases the recall and hence the results’ expressiveness. Furthermore, records in which significant influencing factors for the severity or fatality are shown, but which are not called risk factors in the title or abstract, cannot be identified by the search strategy. An example of this is [53]. It must be assumed that other risk factors for serious and fatal injuries and publications on them exist which are not covered in this review. The studies found only took place in four countries meaning ethnic differences in the course of the disease cannot be considered. A certain bias can also arise from the timing of the search. The search was last updated on 21.04.2020, so that rather early publications are to be expected.

### Comparison with official sites

Table 4 shows the risk factors for severe disease courses form different public authorities. The Robert Koch Institute is Germany’s leading Public Health facility, whereas the Johns Hopkins University is one of the world’s leading facilities for Covid-19 updates. Furthermore, risk factors declared by the United States’ Centers for Disease Control and Prevention and the National Health Service of the United Kingdom are shown. The lists of the different institutions largely overlap. High age (from about 60 years), heart, renal, liver and respiratory diseases as well as diabetes and obesity are frequently mentioned factors. Other factors mentioned include immune compression, male sex, organ receptivity, pregnancy, smoking, secondary diseases, such as cancer or conditions affecting the brain or nerves, and African American ethnicity.

**Table 4:** Overview of risk factors reported by leading institutions

Robert Koch Institute [54]	U.S. CDC [55]	Johns Hopkins Medicine [56]	NHS UK [57]
Higher age (increase from 50–60 years)	Higher age (increase from 65 years)	Higher age (increase from 65 years)	Higher age (increase from 70 years)
Heart diseases	Living in a nursing home or long-term care facility	Diabetes	Organ transplant recipients
Diabetes	Chronic lung disease	Male gender	Lung diseases
Diseases of the respiratory system	Asthma	USA: obesity (BMI ≥ 30)	Blood or bone marrow cancer
Liver diseases	Heart diseases	USA: African American ethnicity	Heart diseases
Renal diseases	Immunosuppression	Comorbidities	Pregnancy
Obesity	Severe obesity (BMI ≥ 40)		Severe obesity (BMI ≥ 40)
Smoking	Diabetes		Chronic kidney diseases
Multimorbidity	Chronic kidney disease undergoing dialysis		Conditions affecting brain or nerves
Immunosuppression	Liver disease		Liver diseases

On the most frequently mentioned points, the risk factors indicated by public authorities coincide with the results of the review. These are liver, heart, renal and respiratory diseases as well as diabetes, obesity, higher age, male gender, comorbidities and even conditions affecting brain and nerves. Risk factors mentioned by public authorities which were not present in this review include multimorbidity, immunosuppression, being an organ transplant recipient, asthma, living in a nursing home, African American ethnicity, blood or bone cancer as well as pregnancy. Even if these could not be confirmed by the review, most of them seem to be very reasonable. Conditions resulting in a diminished immune system such as cancer, immunosuppression or being an organ transplant recipient weaken the body's own immune response to SARS-CoV-2. Another factor is expected to be the prevalent viral pressure, which is high in places where many partly immune-deficient people share little space such as nursing homes. Although studies from the USA were included, no justification for African American ethnicity being a risk factor was found in this review.

This review identified some risk factors not mentioned by public authorities. Mostly these are waiting time to hospital admission, tuberculosis, inflammation disorders and coagulation factors. It is possible that for these factors, especially coagulation factors, not enough evidence is present yet to be support by public authorities.

## Conclusion

Most of the 28 records included in this review describe studies conducted in China. However, regarding the number of patients Italy is outstanding. Conditions and comorbidities potentially connected to a poor state of health such as high age, obesity, diabetes and hypertension were identified as risk factors for severe and fatal disease courses. It was found that severe and even more fatal courses of disease are associated with organ damages mainly affecting the heart, the liver and the kidneys. Further, inflammation and coagulation dysfunctionality were identified as risk factors. For coagulation factors, laboratory values were significantly different in Covid-19 patients but were mostly not mentioned as risk factors in the records’ texts. A prospective study with 12 deceased Covid-19 patients supports this finding. Therefore, the influence of coagulation disorders developed during a SARS-CoV-2 infection should be further investigated.

## Data Availability

All data used is publicly available either by PubMed or CORD-19.

## Code availability

The python code used for preprocessing and search in CORD-19 will be made available upon request.
